# Transforming Commercial Copper Sulfide into Injectable Hydrogels for Local Photothermal Therapy

**DOI:** 10.3390/gels8050319

**Published:** 2022-05-20

**Authors:** Xiaoran Wang, Zizhen Yang, Zhaowei Meng, Shao-Kai Sun

**Affiliations:** 1 Department of Nuclear Medicine, Tianjin Medical University General Hospital, Tianjin 300052, China; mwxiaoran@126.com (X.W.); yang_zizhen@163.com (Z.Y.); 2 School of Medical Imaging, Tianjin Medical University, Tianjin 300203, China

**Keywords:** commercial copper sulfide (CuS), alginate, hydrogel, photothermal therapy (PTT), near-infrared II windows

## Abstract

Photothermal therapy (PTT) is a promising local therapy playing an increasingly important role in tumor treatment. To maximize PTT efficacy, various near-infrared photoabsorbers have been developed. Among them, metal sulfides have attracted considerable interest due to the advantages of good stability and high photothermal conversion efficiency. However, the existing synthesis methods of metal-sulfide-based photoabsorbers suffer from the drawbacks of complicated procedures, low raw material utilization, and poor universality. Herein, we proposed a flexible, adjustable strategy capable of transforming commercial metal sulfides into injectable hydrogels for local PTT. We took copper sulfide (CuS) as a typical example, which has intense second-window near-infrared absorption (1064 nm), to systematically investigate its in vitro and in vivo characteristics. CuS hydrogel with good syringeability was synthesized by simply dispersing commercial CuS powders as photoabsorbers in alginate-Ca^2+^ hydrogel. This synthesis strategy exhibits the unique merits of an ultra-simple synthesizing process, 100% loading efficiency, good biocompatibility, low cost, outstanding photothermal capacity, and good universality. The in vitro experiments indicated that the hydrogel exhibits favorable photothermal heating ability, and it obviously destroyed tumor cells under 1064 nm laser irradiation. After intratumoral administration in vivo, large-sized CuS particles in the hydrogel highly efficiently accumulated in tumor tissues, and robust local PTT was realized under mild laser irradiation (0.3 W/cm^2^). The developed strategy for the synthesis of CuS hydrogel provides a novel way to utilize commercial metal sulfides for diverse biological applications.

## 1. Introduction

Local therapy has recently attracted an increasing amount of attention in tumor treatment due to the advantages of solid selectivity, controllability, and minor systemic side effects [1,2]. Local therapy mainly includes local ablation (radiofrequency ablation [3,4,5], irreversible electroporation [6,7,8], high-intensity focused ultrasound [9], local laser ablation [10], cryoablation [11,12,13], and chemical ablation [14,15]), local phototherapy (PTT [16,17] and photodynamic therapy [16,18,19]), local radioisotope therapy [20,21], local radiotherapy [22,23,24], and local chemotherapy [25]. An increasing number of studies have shown that local therapy can be applied to many clinical situations, such as in the preservation of tissue and function [26], situations without medical indication for surgery [27], the local treatment of metastatic tumors [28,29], and the salvage treatment of recurrent tumors [30]. Thus, local therapy is sometimes essential to improve the quality of life of patients with tumors, as well as survival time. At the same time, some studies have reported the success of the combination of local therapy and other treatment methods, such as immunotherapy [31,32], which further illustrates the broad prospect of local therapy.

As a kind of burgeoning local therapy, PTT uses photoabsorbers to transform near-infrared (NIR) light energy to heat energy, which induces tumor cell necrosis [16,33]. NIR-based PTT is mainly conducted in two biological windows: the first NIR window (NIR-I) and the second NIR window (NIR-II). The wavelength range of NIR-I is from 750 nm to 1000 nm, and the range of NIR-II is from 1000 nm to 1350 nm [34]. Compared with the widely studied NIR-I light, NIR-II light has a stronger penetrating ability and a higher thermal safe power because of its low absorption and scattering in tissue, which has a substantial superiority in curing deeper tumor tissues [34,35,36,37]. In addition to the flourishing development of PTT in fundamental studies [38,39], it is very encouraging that PTT based on local administration has also successfully entered a clinical trial for the treatment of prostate cancer [1], which demonstrates the great potential of PTT-based local therapy in clinical transformation.

To date, plenty of biomaterials have been developed as photoabsorbers, such as organic dyes [40,41,42], organic nanoparticles [43,44,45], noble metal materials [46,47,48], carbon materials [49,50], black phosphorus [51,52], and metal oxide and sulfides [53,54]. Among them, metal sulfides, such as CuS, Bi_2_S_3_, WS_2_, CoS, NiS, and FeS, have high photothermal conversion efficiency because of the surface plasmon resonance effect [55,56], and they have been widely used in PTT in recent years [57,58,59,60,61,62]. In particular, CuS, which possesses strong absorption in the NIR-II bio-window, has been extensively used in NIR-II PTT [63,64]. The current photoabsorbers are mainly obtained using either bottom-up methods or top-down methods, such as coupling thermal oxidation etching and liquid exfoliation to form a solvent-dispersible system [65,66]. However, these methods face several common problems, such as complex steps, high time and energy costs, low raw material utilization, and lacking universal strategy [67].

To avoid the use of metal sulfides using complex synthesis methods, commercial metal sulfides are an excellent choice. The advantage of commercial metal sulfides is that they are mature industrial products with reasonable quality control and low cost, but their disadvantages lie in the raw materials having large particles, being insoluble in water, and not being able to be used for biological applications. Recently, our group proposed a smart “turning solid into gel” strategy [68] by dispersing solid materials in alginate–Ca^2+^ hydrogel (ACH), which can transform solid materials into an injectable hydrogel, making the solid materials bioavailable. Therefore, it is fascinating to develop versatile commercial metal-sulfide-based hydrogels as novel photoabsorbers without complex synthesis.

**Figure 1:**
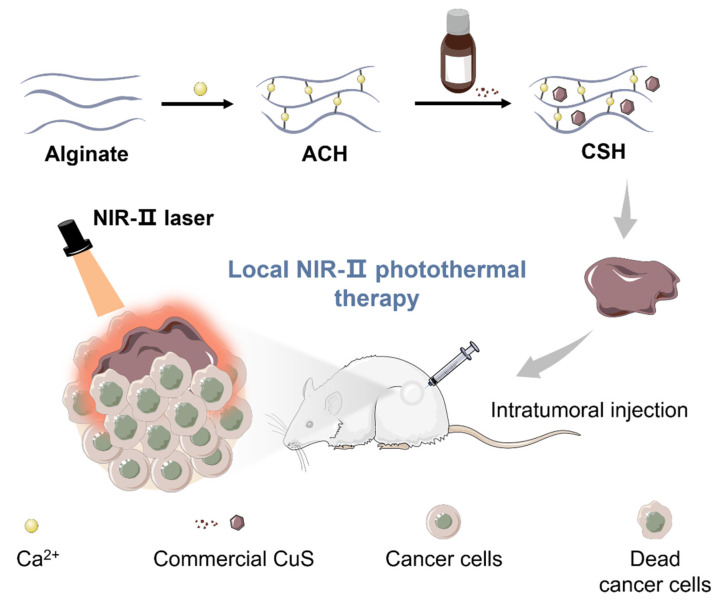
Schematic representation of the synthesis of CSH as a PTT agent for local NIR-II PTT in vivo.

Herein, we introduced a simple and powerful ACH platform to load commercial CuS as a representative sample for local tumor NIR-II PTT (Figure 1). The ultra-simple synthesis, 100% loading efficiency, good biocompatibility, low cost, outstanding photothermal capacity, and extreme flexibility allow this platform to provide more options for highly efficient PTT. The CuS hydrogel (CSH) can be simply obtained through mixing and stirring steps. In vitro experiments indicated that CSH exhibits good syringeability and intense NIR-II absorption (1064 nm). Then, CSH was employed for in vivo PTT studies. The results confirm that this hydrogel not only performs well in killing tumor cells under mild laser irradiation but that it also shows low toxicity in vitro and in vivo. To the best of our knowledge, this is the first time that commercial CuS was elegantly employed for highly efficient PTT in vivo.

## 2. Results and Discussion

### 2.1. Synthesis and Characterization of CSH

**Figure 2:**
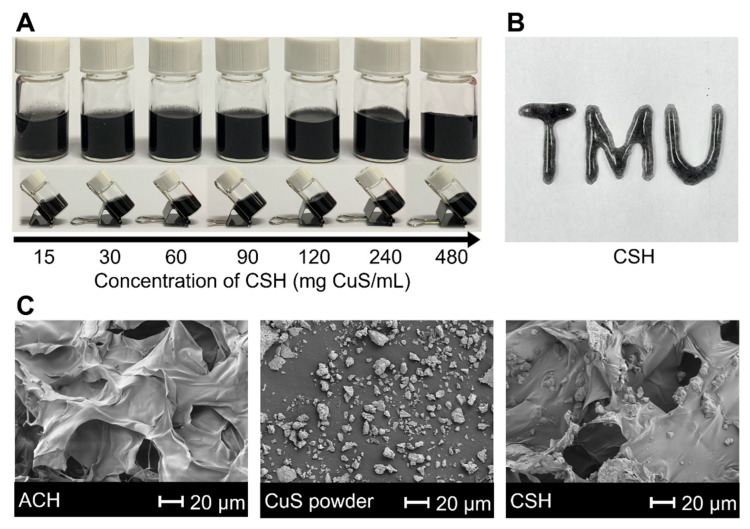
(**A**) Standing and oblique photos of different concentrations of CSH taken immediately after the preparation. (**B**) “TMU” formed by CSH (20 mg CuS/mL) through a 0.45 mm syringe needle. (**C**) SEM images of ACH, CuS particles, and CSH (20 mg CuS/mL).

Firstly, alginate solution and Ca^2+^ were mixed to produce ACH within 1 min based on their strong coordination interaction (Figure S1). Then, CSH was obtained by dispersing commercial CuS powder into ACH. To investigate the loading capacity of ACH, increasing concentrations of CSH were employed. The maximum loading capacity was 480 mg CuS/mL (Figure 2A). The long-term stability of CSH was also monitored (Table S1 and Figure S2). All concentrations of CSH were stable for more than 4 days, and concentrations of 90 mg CuS/mL and below were still stable after 14 days. Considering that excellent syringeability is essential to potential biological applications, we investigated the maximum loading capacity of CSH capable of fluently being injected with different diameters of syringe needles. The results showed that the maximum injectable concentrations for 0.45, 0.5, 0.6, and 1.2 mm syringe needles were 120, 240, 480, and 480 mg CuS/mL, respectively, and a “TMU” pattern could be written by a 0.45 mm syringe with 20 mg CuS/mL CSH (Figure 2B), which proved its excellent syringeability due to the shear-dependent and reversible gel–sol transition (Figure S3) [69].

Rheological experiments showed that the storage moduli (G’) of ACH and CSH were higher than their loss moduli (G’’), demonstrating that ACH and CSH were in a gel state with a relatively weak mechanical strength and flexible shape, which made them easily injectable (Figure S4). As the essential components of CSH, the swelling ratio and degradation behavior of ACH were further investigated. The swelling test showed that ACH could reach swelling equilibrium in 10 min in PBS (pH = 7.4). The swelling ratio of ACH was as high as 13,342.6% (Figure S5), which suggested that the internal cross-linking points of ACH were relatively few, the cross-linking density was low, and the water absorption capacity was strong. According to the ACH degradation curve (Figure S6), the degradation rate of ACH in PBS (pH = 7.4) was 51% after 7 days, which showed its excellent degradability.

The scanning electron microscope (SEM) images of ACH, CSH, and commercial CuS powder were characterized, and they indicated that the CuS particles were dispersed in the ACH with a porous structure (Figure 2C).

### 2.2. Photothermal Performance of CSH In Vitro

**Figure 3:**
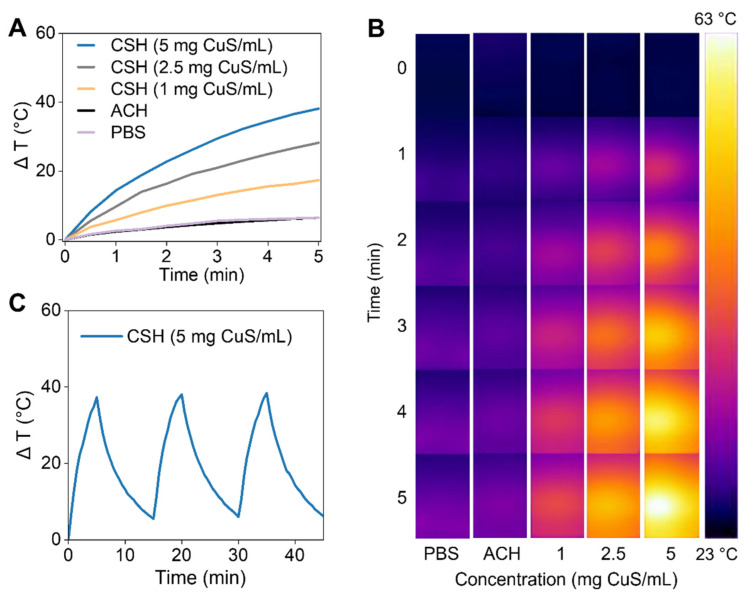
(**A**) Photothermal heating curves of PBS, ACH, and CSH irradiated by NIR-II laser (1064 nm, 1 W/cm^2^). (**B**) The thermal images of PBS, ACH, and CSH under NIR-II laser irradiation taken by an infrared thermal camera. (**C**) The photothermal stability of CSH.

To evaluate the photothermal efficiency of CSH in vitro, different concentrations of CSH were treated with NIR-II laser irradiation (1064 nm, 1 W/cm^2^) for 5 min, and an infrared thermal camera was used to record the temperature elevations. Under NIR-II laser irradiation, CSH showed good photothermal capacity (Figure 3A). The temperature enhancement of CSH with different concentrations increased from 17.3 °C to 38.1 °C, while the temperature increase of ACH and PBS was just 6.4 °C. The thermal images also demonstrate the outstanding photothermal ability of CSH (Figure 3B). After undergoing the heating–cooling process three times, the heating capacity of CSH did not significantly change (Figure 3C), which indicates that CSH has good photothermal stability under NIR-II laser irradiation. Therefore, not only can the prepared CSH efficiently transform NIR laser energy to heat energy, but it can also remain stable after repeated laser illumination.

### 2.3. Cytotoxicity and Cellular Uptake of CSH

**Figure 4:**
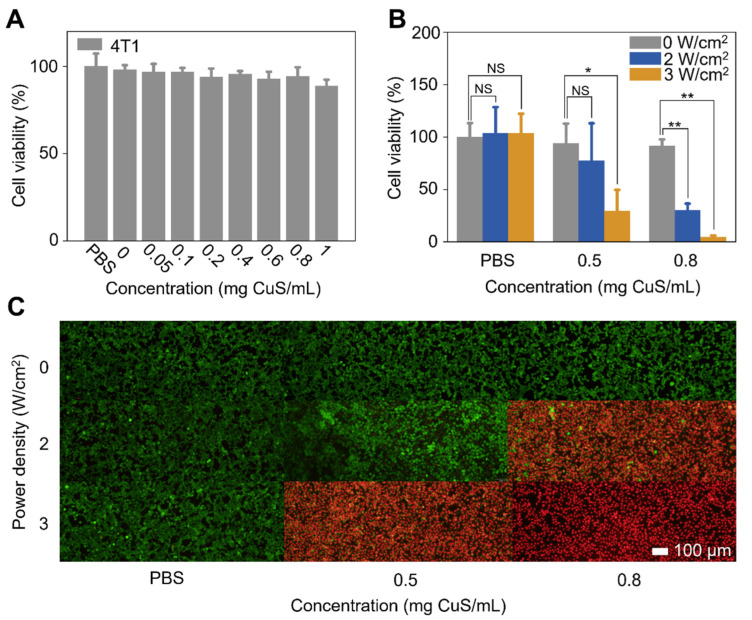
(**A**) Cytotoxicity of 4T1 cells incubated with different concentrations of CSH or PBS. (**B**) Viability of 4T1 cells incubated with PBS or CSH (0.5, 0.8 mg CuS/mL) and irradiated by NIR-II laser (1064 nm: 0, 2, or 3 W/cm^2^) (* *p* < 0.05, ** *p* < 0.01). (**C**) Dead/live cell staining test of 4T1 cells treated with PBS or CSH (0.5, 0.8 mg CuS/mL) and irradiated by NIR-II laser (1064 nm: 0, 2, or 3 W/cm^2^).

CSH, which had a great photothermal efficacy under 1064 nm irradiation, super-large loading capacity, and excellent stability, was capable of being used for further studies. To evaluate its cytotoxicity, different concentrations of CSH were added to 4T1 cells in 96-well plates, and the cells were continued to be cultured for 24 h. Then, the cell viabilities were calculated through a standard MTT assay [3-(4,5-Dimethylthiazol-2-yl)-2,5-diphenyltetrazolium bromide, MTT]. The cell viability was as high as 88.8% after incubation with a high concentration of CSH (1 mg CuS/mL), which indicated the low cytotoxicity of CSH (Figure 4A). The cellular uptake experiment proved that CuS particles in CSH could not be uptaken by cells due to their big size (Figure S7), which illustrates that the mechanism of PTT based on CHS is deduced heat conduction instead of the direct interaction of cell and CSH.

### 2.4. In Vitro PTT of CSH

Due to the low cytotoxicity of CSH, PTT of 4T1 cells using the hydrogel was investigated with an MTT assay and live and dead cell staining. As the standard procedure, 4T1 cells were cultured in a 96-well plate at 37 °C for 24 h, and different concentrations of CSH (0.5 and 0.8 mg CuS/mL) or PBS were added and incubated with the cells at 37 °C. After 1 h, the cells were irradiated by a 1064 nm laser (0, 2, or 3 W/cm^2^) for 5 min. The 4T1 cell viabilities showed CSH-concentration- and laser-power density-dependent deceases. After being treated with both 0.8 mg CuS/mL of CSH and 1064 nm laser irradiation (3 W/cm^2^), 4T1 cell viability dropped to less than 4%. However, the viability of 4T1 cells treated with only CSH or laser irradiation remained approximately 100% (Figure 4B). The fluorescent images of live and dead cells, which were stained by calcein acetoxymethyl ester (calcein AM) and propidium iodide (PI), respectively, also showed that the 4T1 cells were significantly destructed after the combined treatments (Figure 4C). These results prove that CSH has an excellent photothermal effect on tumor cells with 1064 nm laser irradiation.

### 2.5. Intratumoral Retention Test of CSH

**Figure 5:**
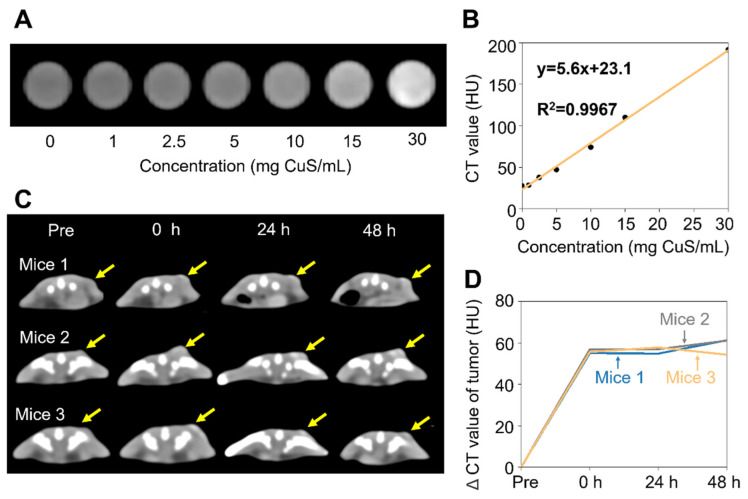
(**A**) CT images of CSH at different concentrations. (**B**) CT value curve of CSH. (**C**) CT scan of BALB/c mice before and 0, 24, and 48 h after being intratumorally injected with 20 mg CuS/mL of CSH (*n* = 3). (**D**) CT value (Hounsfield, HU) changing curves on the tumor site of BALB/c mice.

In order to assess the intratumoral retention ability of CSH, computer tomography (CT) scans were carried out in vitro and in vivo under a voltage of 120 kV (clinical use). Although CSH was considered to have a weak CT value attenuation (Figure 5A,B), after intratumoral injection, it could still be found at the tumor site with CT scans. During the 2 days of CT monitoring that followed, no significant change was found in the CT value or in the morphology of CSH (Figure 5C,D), proving its good retention ability at tumor sites.

### 2.6. In Vivo PTT of CSH

**Figure 6:**
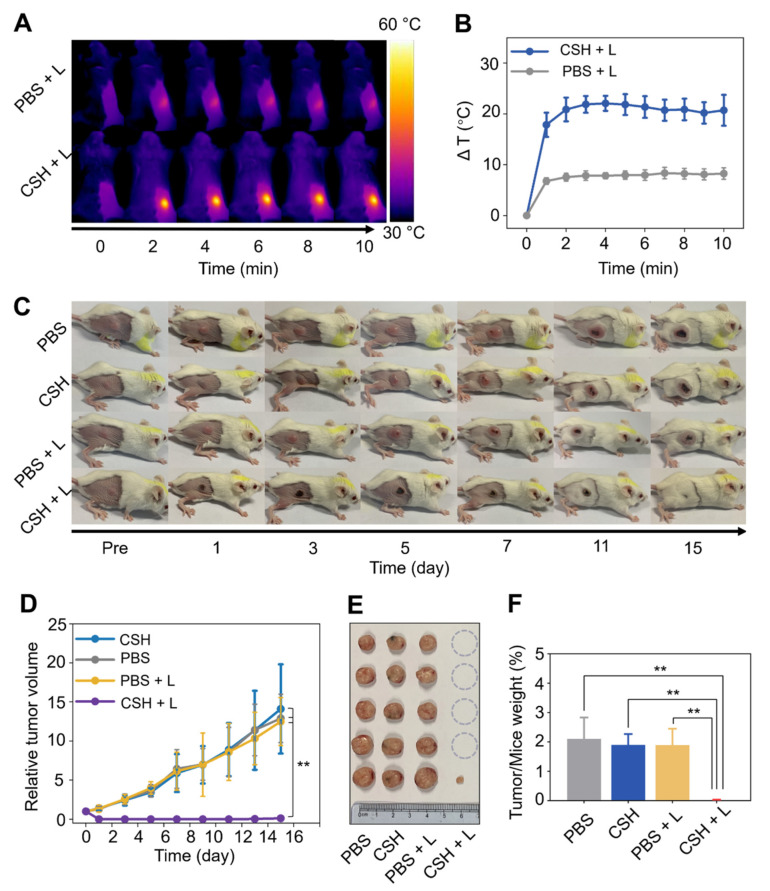
(**A**) Thermal images of tumor-bearing mice treated with only PBS or both CSH and NIR-II laser irradiation. (**B**) Photothermal heating curves of tumor sites taken with an infrared thermal camera. (**C**) Tumor-monitoring photography of mice in various groups. (**D**) Relative tumor volume curves of mice in different groups (*n* = 5 in each group, ** *p* < 0.01). (**E**) Excised tumors from the mice on the 15th day of the observation period. (**F**) Ratio of final tumor weight to final body weight of mice in different groups (** *p* < 0.01).

To minimize the damage to surrounding tissues, a mild laser power (0.3 W/cm^2^) was employed for in vivo PTT. CSH with a concentration of 20 mg CuS/mL, which can cause a significant temperature rise in vitro, was used to guarantee effective tumor ablation (Figure S8). To evaluate the in vivo photothermal tumor therapy efficacy of CSH in the NIR-II bio-window (1064 nm), BALB/c mice were grouped according to different treatments (*n* = 5) as follows: (1) only PBS; (2) only CSH; (3) PBS + laser; and (4) CSH + laser. In comparison with the control, there was a noticeable temperature increase at the tumor site after being injected with CSH and irradiated with a 1064 nm laser (Figure 6A,B). Tumor sizes were measured every 2 days to evaluate the anti-tumor capacity. The results showed that the tumor growth of mice treated with both CSH and laser irradiation was effectively inhibited, and the tumors were eliminated after PTT (Figure 6C). There was tumor recurrence in only one mouse in the combined treatment group, and the recurred tumor was significantly smaller than that in the other groups. In contrast, the tumors in the other groups multiplied, and the final tumor volumes after 15 days of growth were about 12.9, 14.1, and 12.5 times larger than the initial tumor volume in groups 1, 2, and 3, respectively (Figure 6D). The tumors were dissected and photographed on the 15th day (Figure 6E). The dissected tumors were weighed, and the ratio of tumor weight to mouse body weight in each group was calculated (Figure 6F), which further revealed that the tumors were obviously suppressed by CSH-based PTT. These results illustrate that CSH can wreck tumors entirely due to its high thermal efficiency in vivo.

### 2.7. In Vivo Toxicity of CSH

**Figure 7:**
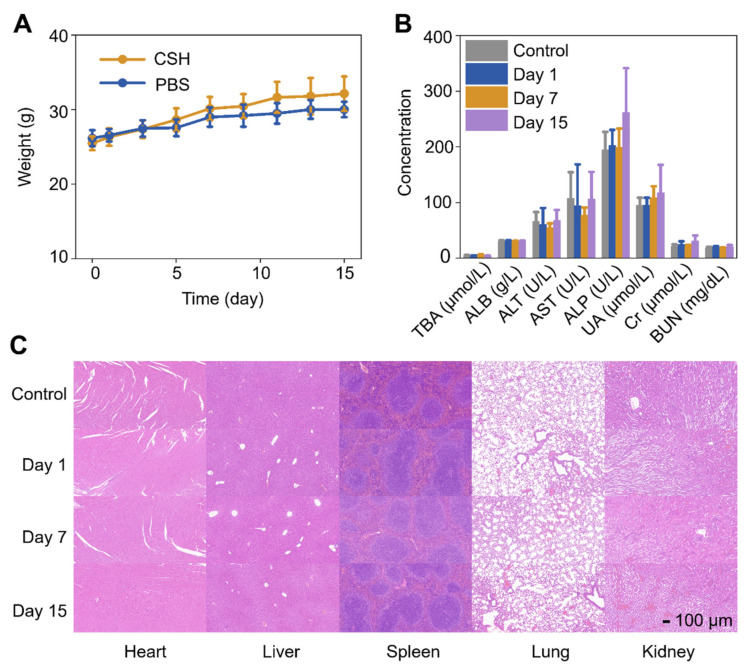
(**A**) Weight-changing curves of mice after being subcutaneously injected with PBS and 20 mg CuS/mL of CSH (*n* = 5 in each group). (**B**) Blood biochemical indexes of mice measured after being treated with PBS for 15 days and CSH (20 mg CuS/mL) for 1, 7, and 15 days (*n* = 5 in each group). (**C**) H&E-stained images of major organs of Kunming mice acquired after being treated with PBS for 15 days and CSH (20 mg CuS/mL) for 1, 7, and 15 days.

To assess the systemic toxicity of CSH in vivo, the weight monitoring, blood biochemistry analysis, and H&E staining of major organs of the mice were accomplished. The weight monitoring results displayed no noticeable difference in body weight among the mice with various treatments (Figure 7A). The blood biochemistry analysis indicated that the liver and kidney function indexes of the mice were entirely within the normal range (Figure 7B), and no evident inflammatory lesion or organ damage was found in all major organs of the mice (Figure 7C). All of the above results confirm that CSH has good biocompatibility, which makes it a promising PTT agent with good biosafety and photothermal efficacy in vivo.

## 3. Conclusions

In conclusion, according to the “turning solid into gel” strategy, a robust metal sulfide hydrogel system was established to load commercial metal sulfide powders for high-efficiency tumor PTT. As a representative metal sulfide, commercial CuS powder was studied in depth. The obtained CSH was verified to have good stability, favorable syringeability, potent photothermal efficacy, and excellent retention capability at the injection site. Due to the deeper tissue penetration of NIR-II light, further studies were investigated using 1064 nm laser irradiation. The follow-up experimentations in vitro and in vivo showed the CSH to have negligible toxicity and a high photothermal killing effect on tumor cells under the irradiation of the 1064 nm laser. Therefore, as a new method of photothermal agent preparation, transforming commercial sulfides into injectable hydrogels can help to save costs, improve accuracy, and raise efficiency without worrying about toxicity, all of which give it great hope for clinical transformation.

## 4. Materials and Methods

### 4.1. Materials

CaCl_2_ and sodium alginate (200 ± 20 mPa s) were obtained from Aladdin Biochemical Technology Co., Ltd. (Shanghai, China). CuS was purchased from Sigma-Aldrich trade Co., Ltd. (Shanghai, China). Fetal Bovine Serum (FBS) was provided by Lanzhou Minhai Bio-Engineering Co., Ltd. Dulbecco’s Modified Eagle Medium (DMEM) was obtained from ThermoFisher Instrument Co., Ltd. (Suzhou, China). MTT was bought from Aladdin Biochemical Technology Co., Ltd. (Shanghai, China). Calcein AM and PI were provided by Dojindo Chemical L.L.C. (Shanghai, China). DMSO was purchased from Concord Technology Co., Ltd. (Tianjin, China). Ultrapure water was bought from Wahaha Group Co., Ltd. (Hangzhou, China).

### 4.2. Synthesis of ACH and CSH

Typically, 0.5 mL of sodium alginate (10 mg/mL) and 0.05 mL of CaCl_2_ (10 mg/mL) were mixed with 0.45 mL H_2_O to prepare ACH. Then, commercial CuS powder was added to ACH, and the system was stirred for 15 min to obtain CSH.

### 4.3. Stability Assessment of CSH

The homogeneous stability of CSH was evaluated for 2 weeks. Briefly, different concentrations of CSH (15, 30, 60, 90, 120, 240, 480 mg CuS/mL) were placed in vials, respectively. If the stability time was less than 10 min or the sulfide could not be dispersed in ACH, the mixture was regarded as overloaded. The stable concentrations of CSH were monitored for 14 days and photographed at different time points (10 min, 20 min, 30 min, 1 h, 2 h, 3 h, 4 h, 6 h, 8 h, 10 h, 12 h, 14 h, 16 h, 18 h, 20 h, 24 h, and then every day). During the monitoring period, if the hydrogel was found to be layered, it was regarded as precipitation.

### 4.4. Syringeability of CSH

The syringeability of CSH was evaluated using four sizes of syringe needles (26 G, 0.45 mm; 25 G, 0.5 mm; 23 G, 0.6 mm; 18 G, 1.2 mm). Different concentrations of CSH were extruded through the various sizes of syringe needles. For every size of syringe, the maximum injectable concentration was recorded, and a “TMU” (an abbreviation of “Tianjin Medical University”) was written by its maximum injectable concentration. Then, a “TMU” was formed by CSH (20 mg CuS/mL) through a 0.45 mm syringe needle, which was used for in vivo PTT.

### 4.5. Characterization

Rheology experiments of ACH and CSH (20 mg CuS/mL) were conducted on a DHR-2 rheometer (TA Instruments), with a strain amplitude of 1% and an angular frequency of 10 rad/s for dynamic oscillatory time sweep measurements.

The swelling ratio and degradation behavior of ACH were investigated according to a previous study [70]. To calculate the swelling ratio, lyophilized ACH was weighed (recorded as M_0_), immersed in PBS (pH = 7.4), and incubated in an incubator shaker at a shaking speed of 100 rpm at 37 °C. The swelled ACH was removed, and the surface water was wiped out. Then, the collected ACH was weighed at a specific time interval (recorded as Mt). The swelling ratio (%, *w*/*w*) was calculated using the equation (M_t_ − M_0_)/M_0_ × 100. The experiments were carried out in triplicate to obtain an average value. The degradation behavior of ACH was assessed in PBS (pH = 7.4). The lyophilized ACH was weighed (recorded as Mi) and completely immersed in PBS, and then it was degraded in an incubator shaker at 37 °C and 100 rpm. After different time intervals (1, 3, or 7 days), ACH was washed with ultrapure water to remove PBS, freeze-dried, and weighed (recorded as M_f_). The degradation rate (%, *w*/*w*) was calculated using the equation (M_i_ − M_f_)/M_i_ × 100.

Field-emission scanning electron microscopy (FE-SEM) images of ACH, CuS powder, and CSH (20 mg CuS/mL) were acquired under a 2 kV accelerating voltage on a Gemini SEM 300 (ZEISS, Germany) microscope.

### 4.6. Photothermal Performance In Vitro

In order to evaluate the photothermal efficacy of CSH in vitro, PBS or different concentrations of CSH (0, 1, 2.5, and 5 mg CuS/mL) with a volume of 1 cm^3^ were placed in cuvettes with a base area of 1 cm^2^. Then, cuvettes were irradiated with a 1064 nm laser (1 W/cm^2^) for 5 min, and temperature elevations were recorded using an infrared thermal camera. In order to test its photothermal stability, CSH (5 mg CuS/mL, 1 mL) was put into a cuvette and irradiated using NIR-II (1064 nm) laser with a power density of 1 W/cm^2^ for 5 min, and then the system was cooled for 10 min to bring the temperature close to room temperature; the process was repeated three times.

### 4.7. Cell Culture and Animals

The growth and metastasis of 4T1 cells in BALB/c mice are similar to those of human breast cancer, making the cells a relatively classical and widely used cell line to test the therapeutic effects on tumors [71]. Therefore, the 4T1 cell line was used to study CSH in vitro and in vivo. 4T1 cells were cultured in a culture medium with 90% DMEM and 10% FBS. Cells were cultured in a humidified incubator (5% CO_2_ and 37 °C), and the culture medium was refreshed at 1–2 day intervals. Kunming mice and BALB/c mice were purchased from Beijing HFK Bioscience Co., Ltd. (Beijing, China). All animal experiments were performed according to the protocols established by the Animal Care and Use Committee of Tianjin Medical University, and all experimental operations were approved by the Animal Care and Use Committee.

### 4.8. Cytotoxicity and Cellular Uptake Assay

To determine the potential cytotoxic effects of CSH, 4T1 cells (1 × 10^4^ per well) were cultured in 96-well plates with 200 μL of cell culture medium per well for 24 h. Then, after the exchange of the cell medium, PBS or different concentrations of CSH (0, 0.05, 0.1, 0.2, 0.4, 0.6, 0.8, 1 mg CuS/mL) were added to the wells. After 24 h incubation, cell viabilities were evaluated via a standard MTT test. After the cells were washed with PBS, new cell medium and MTT (10 μL, 5 mg/mL) were added and incubated with the cells for 4 h; then, the supernatant was discarded, and 120 μL of DMSO per well was added. Finally, the wells’ absorptions at 490 nm were measured using a microplate reader.

The cellular uptake mechanism of CSH was also investigated. In brief, 4T1 cells (1 × 10^4^ per well) were cultured in 96-well plates. After 24 h, PBS or different concentrations of CSH (0, 0.05, 0.1, 0.2, 0.4, 0.6, 0.8, 1 mg CuS/mL) were added and co-incubated with the cells for another 24 h. Then, the cells were washed with PBS, and 120 μL of PBS per well was added. Finally, the cells were observed under a microscope.

### 4.9. In Vitro Photothermal Cytotoxicity Study

The photothermal cell killing ability of CSH under 1064 nm laser irradiation was evaluated using the MTT assay. 4T1 cells (1.4 × 10^4^ per well) were incubated in a 96-well plate for 24 h. After being washed with PBS, the 4T1 cells were treated with PBS or different concentrations of CSH (0.5 or 0.8 mg CuS/mL) for 1 h, and they were irradiated with varying densities of power of 1064 nm laser (0, 2, or 3 W/cm^2^) for 5 min. Then, cell viabilities were measured using the MTT assay, and the absorption of each well at 490 nm was recorded using a microplate reader.

### 4.10. Live/Dead Cells Staining Test

To further investigate the PTT efficacy in vitro, 4T1 cells (1.4 × 10^4^ per well) were incubated in 96-well plates for 24 h, and CSH (0.5, 0.8 mg CuS/mL) or PBS was added and co-incubated with 4T1 cells for 1 h. Then, 4T1 cells were exposed to NIR-II laser (1064 nm, 0, 2, or 3 W/cm^2^) for 5 min, thoroughly washed with PBS twice, and stained with calcein AM and PI. Fluorescent images were recorded with an inverted luminescence microscope.

### 4.11. Intratumoral Retention Test of CSH

In vitro and in vivo CT scans were carried out via a clinical X-ray CT (SOMATOM Force, Siemens healthineers, Erlangen, Germany) under a clinical voltage (120 kV) [72]. CSHs with different concentrations (0, 1, 2.5, 5, 10, 15, 30 mg CuS/mL) were prepared, and then CT images of CSH were collected. For in vivo CT imaging, 50 μL of CSH (20 mg CuS/mL) was intratumorally injected into BALB/c mice (*n* = 3). Then, the mice were scanned pre-injection and after injection at different time points (0 h, 24 h, and 48 h). CT values were measured using Radiant DICOM Viewer software.

### 4.12. Anti-Tumor Assessment In Vivo

To ensure biosafety, a mild laser power (0.3 W/cm^2^) and CSH with a concentration of 20 mg CuS/mL were used for in vivo PTT. To verify the in vitro heating effect, CSH (20 mg CuS/mL) or PBS was made into 50 μL droplets, and they were irradiated using a 1064 nm laser (0.3 W/cm^2^) for 5 min. Thermal images of them were taken, and photothermal heating curves were obtained. Then, to explore the anti-tumor ability of CSH with 1064 nm laser irradiation, tumor-bearing BALB/c mice were divided into 4 groups (*n* = 5) as follows: (1) only PBS, (2) only CSH, (3) PBS + laser, and (4) CSH + laser. Mice in Group 1 were intratumorally injected with PBS (50 μL). Mice in Group 2 were intratumorally injected with CSH (20 mg CuS/mL, 50 μL). Mice in Group 3 were intratumorally injected with PBS and exposed to 1064 nm laser irradiation (0.3 W/cm^2^) for 10 min. Mice in Group 4 were intratumorally injected with CSH (20 mg CuS/mL, 50 μL) and exposed to 1064 nm laser irradiation (0.3 W/cm^2^) for 10 min. The hyperthermia effect on tumor site was carefully recorded using an infrared thermal camera. Then, tumor sizes were measured and recorded every 2 days. Tumor volume was calculated using the following formula: V = a × b^2^/2, where a and b mean the longest and shortest diameters, respectively. The relative volume of the tumors was the ratio of the day’s volume to the initial volume. Photos of tumors in all groups were taken every 2 days, and the tumors were removed and weighed on day 15 after the treatment.

### 4.13. Statistics

The differences between groups were studied using one-way ANOVA, and “*p*” value < 0.05 was considered as statistically significant. All analyses were conducted using GraphPad Prism 8.0.2 software.

### 4.14. In Vivo Biosafety Analysis

To evaluate the biosafety of CSH in vivo, weight monitoring, blood biochemistry analysis, and H&E staining were conducted on Kunming mice. To monitor the body weight change, CSH (50 μL, 20 mg CuS/mL) or PBS was subcutaneously injected into Kunming mice (*n* = 5, respectively), and their body weights were recorded every two days until the 15th day. For blood biochemistry analysis and H&E staining, Kunming mice were subcutaneously injected with PBS (*n* = 5) or CSH (50 μL, 20 mg CuS/mL) (*n* = 15). Mice in the hydrogel-injected group were dissected on the 1st, 7th, and 15th days (*n* = 5 every time), and mice in the PBS group were dissected on the 15th day. After the mice were dissected, their major organs (i.e., heart, lung, spleen, liver, and kidney) were removed and stained with hematoxylin and eosin, and blood samples were collected. The blood samples were centrifugated at 3000 rpm for 10 min to separate and collect the supernatant serum. Then, the blood biochemistry biomarkers were analyzed, which included albumin (ALB), total bile acid (TBA), aspartate aminotransferase (AST), alanine aminotransferase (ALT), and alkaline phosphatase (ALP) for liver function assessment, and uric acid (UA), urea nitrogen (BUN), and serum creatinine (Cr) for kidney function evaluation.

## Data Availability

Not applicable.

## Supplementary Materials

The following supporting information can be downloaded at: https://www.mdpi.com/article/10.3390/gels8050319/s1, Figure S1: Photos of ACH stirred for different times after mixing alginate solution and Ca^2+^ solution; Figure S2: Standing and oblique photos of different concentrations of CSH taken on day 7 and day 14; Figure S3: The maximum injectable concentration of CSH through various size of syringes and “TMU” written by them; Figure S4: Rheological properties of ACH and CSH (20 mg CuS/mL); Figure S5: Curve of swelling ratio of ACH in PBS (pH = 7.4); Figure S6: Degradation curve of ACH in PBS (pH = 7.4); Figure S7: Photos of 4T1 cells incubated with different concentrations of CSH or PBS for 24 h; Figure S8: Thermal images and photothermal heating curves of CSH (20 mg CuS/mL) and PBS under 1064 nm laser irradiation in vitro; Table S1: The stability of different concentrations of CSH.
