# Supplementary material for: Transforming Commercial Copper Sulfide into Injectable Hydrogels for Local Photothermal Therapy
Source: Gels. 2022 May 20;8(5):319. doi: 10.3390/gels8050319 (PMC9141692; doi:10.3390/gels8050319)
Supplement: Supplementary file 1 [file gels-08-00319-s001.zip › gels-1604424-supplementary.pdf]

gels

Supplementary Materials

Transforming Commercial Copper Sulfide into Injectable Hydrogels for Local Photothermal Therapy

Xiaoran Wang [1,†], Zizhen Yang [1,†], Zhaowei Meng [1,*] and Shao-Kai Sun [2,*]

Table S1. The stability of different concentrations of CSH.

Concentration (mg CuS/mL)	15	30	60	90	120	240	480
settling time (days)	>14	>14	>14	>14	12	9	5

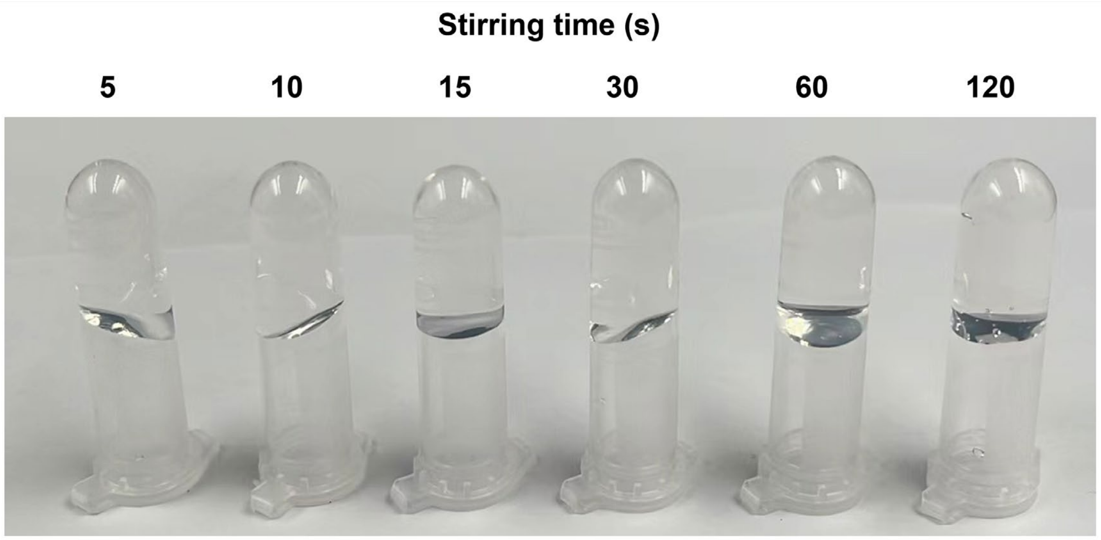

Figure S1. Photos of ACH stirred for different times after mixing alginate solution and Ca^{2+} solution.

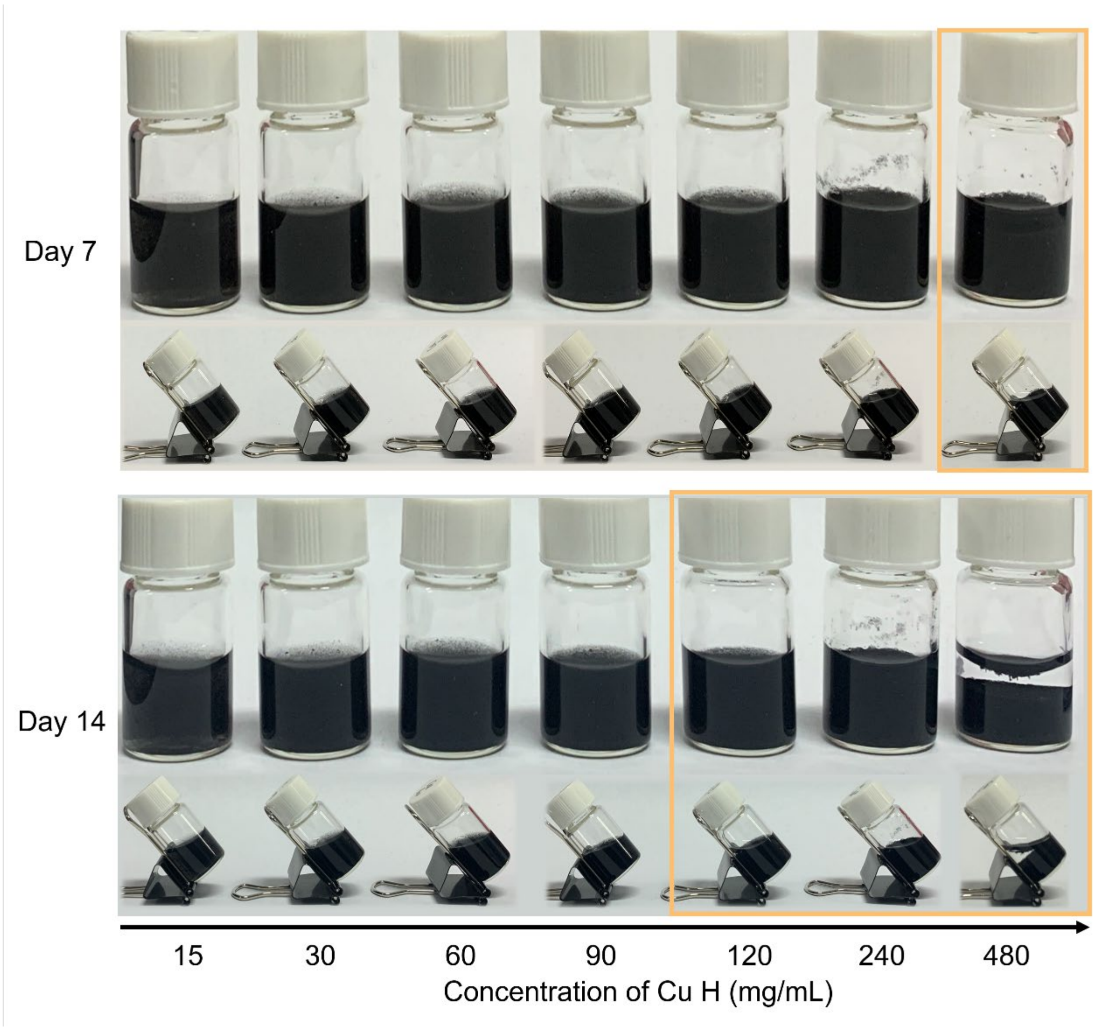

Figure S2. Standing and oblique photos of different concentrations of CSH taken on day 7 and day 14.

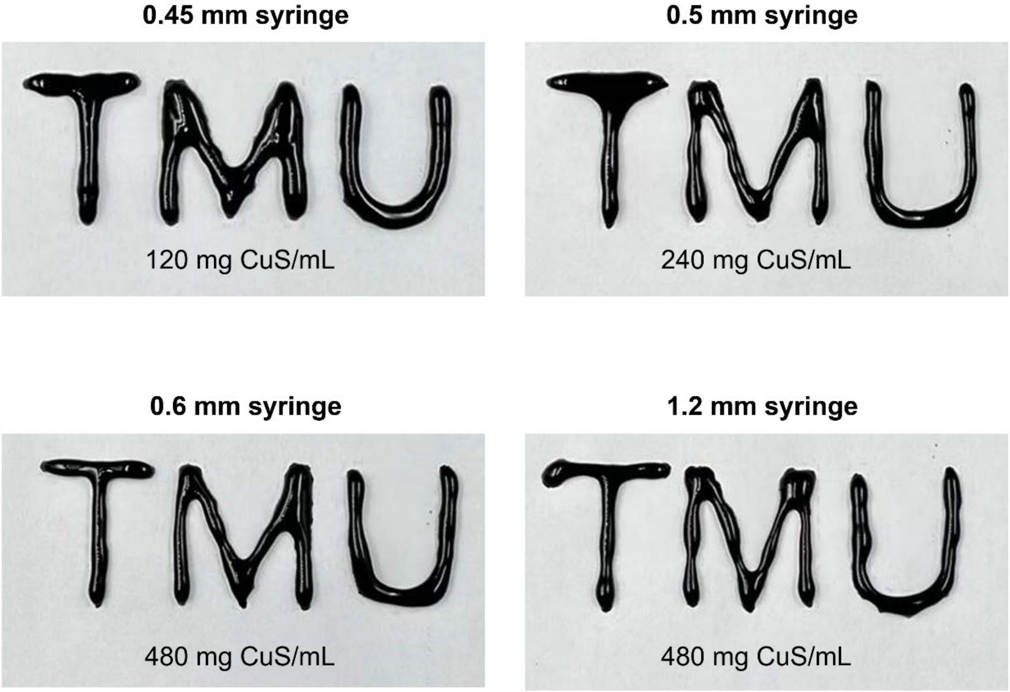

Figure S3. The maximum injectable concentration of CSH through various size of syringes, and "TMU" written by them.

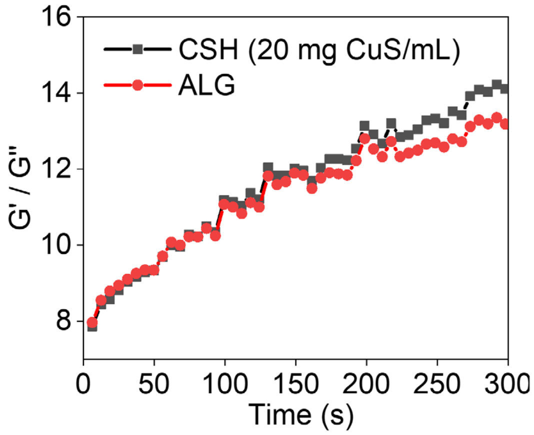

Figure S4. Rheological properties of ACH and CSH (20 mg CuS/mL). G': storage modulus, G'': loss modulus.

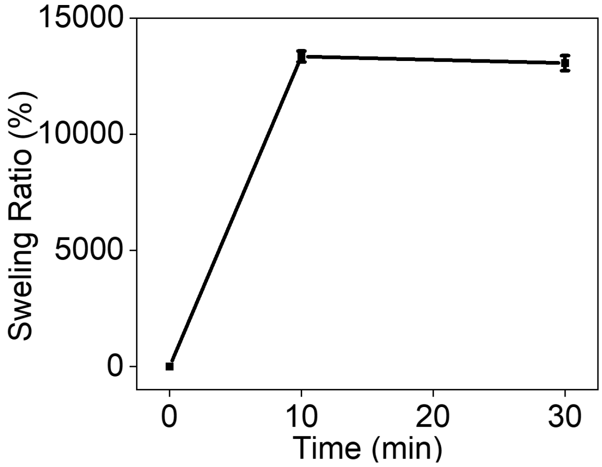

Figure S5. (A) Curve of swelling ratio of ACH in PBS (pH=7.4).

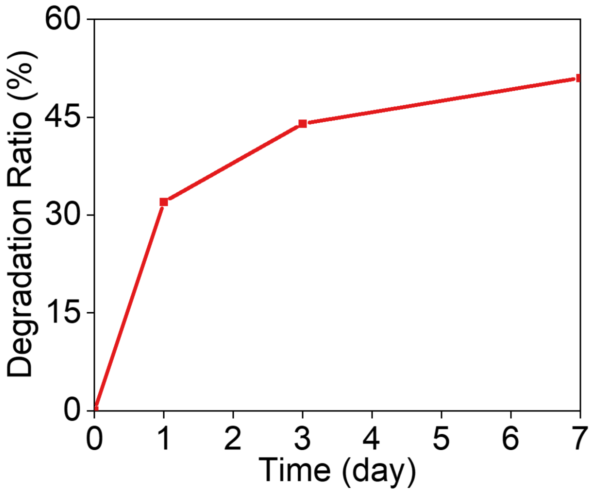

Figure S6. Degradation curve of ACH in PBS (pH=7.4).

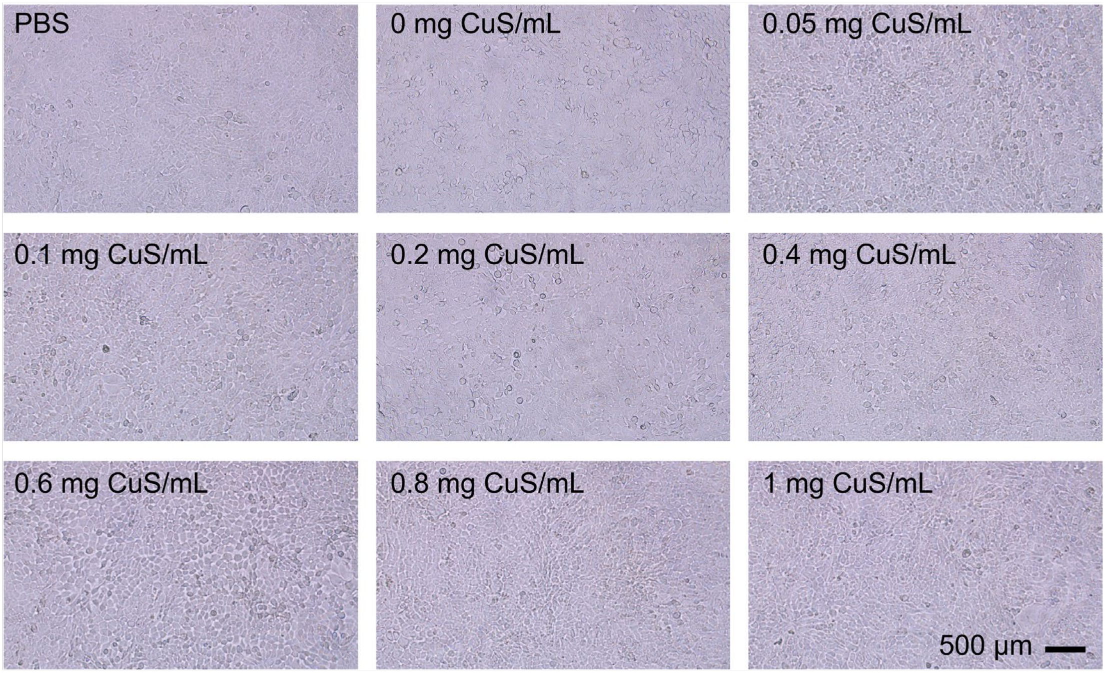

Figure S7. Photos of 4T1 cells incubated with different concentrations of CSH or PBS for 24 h.

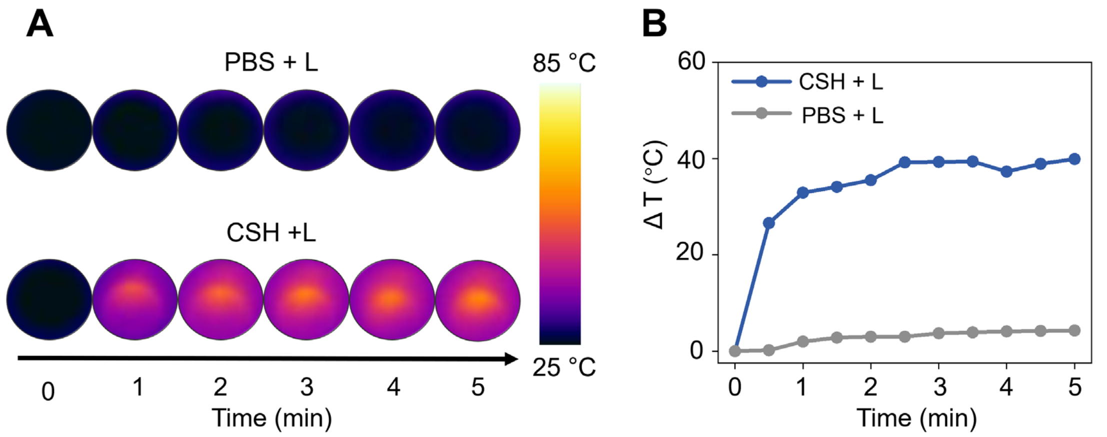

Figure S8. Thermal images (A) and photothermal heating curves (B) of CSH (20 mg CuS/mL) and PBS under 1064 nm laser irradiation in vitro.
